# Effects of resistance training on postural control in Parkinson’s disease: a randomized controlled trial

**DOI:** 10.1590/0004-282X-ANP-2020-0285

**Published:** 2021-06-30

**Authors:** Janini CHEN, Hsin Fen CHIEN, Debora Cristina Valente FRANCATO, Alessandra Ferreira BARBOSA, Carolina de Oliveira SOUZA, Mariana Callil VOOS, Julia Maria D'Andréa GREVE, Egberto Reis BARBOSA

**Affiliations:** 1 Universidade de São Paulo, Hospital das Clínicas, Faculdade de Medicina, Departamento de Neurologia, Clínica de Distúrbios do Movimento, São Paulo SP, Brazil. Universidade de São Paulo Hospital das Clínicas Faculdade de Medicina São Paulo SP Brazil; 2 Grupo de Pesquisa em Reabilitação em Distúrbios do Movimento, São Paulo SP, Brazil. Grupo de Pesquisa em Reabilitação em Distúrbios do Movimento São Paulo SP Brazil; 3 Universidade de São Paulo, Hospital das Clínicas, Faculdade de Medicina, Departamento de Ortopedia e Traumatologia, São Paulo SP, Brazil. Universidade de São Paulo Hospital das Clínicas Faculdade de Medicina São Paulo SP Brazil; 4 Universidade de São Paulo, Departamento de Fisioterapia, Terapia Ocupacional e Fonoaudiologia, São Paulo SP, Brazil. Universidade de São Paulo Departamento de Fisioterapia Terapia Ocupacional e Fonoaudiologia São Paulo SP Brazil; 5 Universidade de São Paulo, Hospital das Clínicas, Faculdade de Medicina, Instituto de Ortopedia e Traumatologia, Laboratório de Estudo do Movimento, São Paulo SP, Brazil. Universidade de São Paulo Hospital das Clínicas Faculdade de Medicina São Paulo SP Brazil

**Keywords:** Resistance Training, Parkinson Disease, Postural Balance, Rehabilitation, Quality of Life, Treinamento de Resistência, Doença de Parkinson, Equilíbrio Postural, Reabilitação, Qualidade de Vida

## Abstract

**Background:**

Postural instability affects Parkinson’s disease (PD) patients’ postural control right from the early stages of the disease. The benefits of resistance training (RT) for balance and functional capacity have been described in the literature, but few studies have been conducted showing its effects on PD patients’ postural control.

**Objective:**

To investigate the effects of a three-month RT intervention on static posturography (SP) measurements and clinical functional balance assessment among PD patients.

**Methods:**

Seventy-four patients were randomly assigned to a three-month RT intervention consisting of using weightlifting machines at a gym (gym group) or RT consisting of using free weights and elastic bands (freew group), or to a control group. The participants were evaluated at baseline, three months and six months. We evaluated changes of SP measurements under eyes-open, eyes-closed and dual-task conditions (primary endpoint), along with motor performance and balance effects by means of clinical scales, dynamic posturography and perceptions of quality of life (secondary endpoints).

**Results:**

There were no significant interactions in SP measurements among the groups. Unified Parkinson Disease Rating Scale (UPDRS-III) motor scores decreased in both RT groups (p<0.05). Better perceived quality of life for the mobility domain was reported in the gym group while functional balance scores improved in the freew group, which were maintained at the six-month follow-up (p<0.05).

**Conclusions:**

This study was not able to detect changes in SP measurements following a three-month RT intervention. Both RT groups of PD patients showed improved motor performance, with positive balance effects in the freew group and better perceived quality of life in the gym group.

## RESUMO

**Introdução:**

A instabilidade postural afeta o controle postural desde os estágios iniciais da doença de Parkinson (DP). A literatura descreve benefícios do programa de fortalecimento muscular no equilíbrio, na capacidade funcional, porém poucos estudos investigaram seus efeitos no controle postural na DP.

**Objetivo:**

Investigar os efeitos de um programa de três meses de fortalecimento nas medidas da posturografia estática (PE) e nos testes clínicos na DP.

**Métodos:**

Participaram do ensaio aleatório controlado 74 pacientes, designados em um dos seguintes grupos: fortalecimento utilizando aparelhos de musculação (gmusc); fortalecimento com pesos livres e elásticos (gpeso); e grupo controle. As avaliações ocorreram antes da intervenção, 3 e 6 meses após intervenção. Foram avaliados alterações nas medidas da PE nas condições de olhos abertos, olhos fechados e dupla tarefa (desfecho primário), efeitos sobre sintomas motores, equilíbrio por meio de escalas clínicas, posturografia dinâmica e percepção da qualidade de vida (desfechos secundários).

**Resultados:**

Não houve interações significativas nas medidas da PE entre os grupos. As pontuações motoras da Escala Unificada de Avaliação da Doença de Parkinson (UPDRS-III) diminuíram em ambos os grupos de fortalecimento (p<0,05). Houve melhora da percepção da qualidade de vida para o domínio mobilidade no gmusc, assim como ganhos nas pontuações dos testes funcionais no gpeso, mantido no seguimento após seis meses (p<0,05).

**Conclusões:**

O programa de fortalecimento não altera o controle postural medida pela PE. Apesar disso, ambos os grupos de fortalecimento apresentaram melhor desempenho motor, efeitos positivos a médio prazo no equilíbrio no gpeso e melhor desempenho na qualidade de vida no gmusc.

## INTRODUCTION

Parkinson’s disease (PD) patients show lack of postural stability and motor coordination^1^ and impaired ability to keep the center of mass over the base of support during movement. Maintaining upright stance involves muscle activation and joint integrity as well as neural responses to external disturbances^2^^,^^3^^,^^4^.

Assessing postural control is challenging, but static posturography (SP) provides quantitative information on postural control. SP measures shifts in the vertical forces on a force platform that are exerted by body sway during upright stance and these measurements make it possible to infer the center of pressure (COP). PD patients have larger COP displacement variability than healthy older adults^5^^,^^6^^,^^7^. Moreover, studies have suggested that there may be an association between mediolateral sway, increased COP velocity, poor postural control and risk of falls in this population^8^^,^^9^.

It has been reported in the literature that muscle strength^10^, mobility^11^ and balance^12^ may improve with resistance exercise training (RT), with a positive impact on functional capacity and reduction of the risk of falls in PD^13^. However, only a few well-designed controlled studies^14^^,^^15^^,^^16^^,^^17^ have qualitatively assessed SP in relation to RT programs, RT modalities and postural control, among PD patients.

Therefore, the aim of this study was primarily to determine the effects of a three-month RT intervention on SP measurements among PD patients. The secondary objective was to evaluate the impact of this exercise intervention on motor performance, functional balance scores, dynamic posturography measurements and perceptions of quality of life (QoL).

## METHODS

### Study design and participants

We conducted a three-arm, single-blind randomized controlled trial. Patients were recruited from the outpatient clinic of the Movement Disorders Clinic, Hospital das Clinicas HCFMUSP, Department of Neurology, Faculdade de Medicina, Universidade de São Paulo, and from the Brazil Parkinson Association, São Paulo, between September 2013 and February 2016.

### Eligibility criteria

The study inclusion criteria were: idiopathic PD diagnosis based on the United Kingdom Parkinson’s Disease Society Brain Bank diagnostic criteria^18^; age 50-75 years; Hoehn and Yahr (HY) stage scores of 2-3; antiparkinsonian drug treatment consisting of stable daily doses for at least three months before inclusion; ability to walk independently without assistance devices; and Mini-Mental State Examination (MMSE) score of 24 or more. The exclusion criteria were: orthopedic conditions; severe pain; unstable cardiovascular and/or metabolic disease; vestibular dysfunction; prior stroke; and attending a physical rehabilitation program at least six months before inclusion.

This study was approved by the local ethics committee and was registered at ClinicalTrials.gov (NCT: 02674724).

### Randomization and blinding

**Figure 1.**
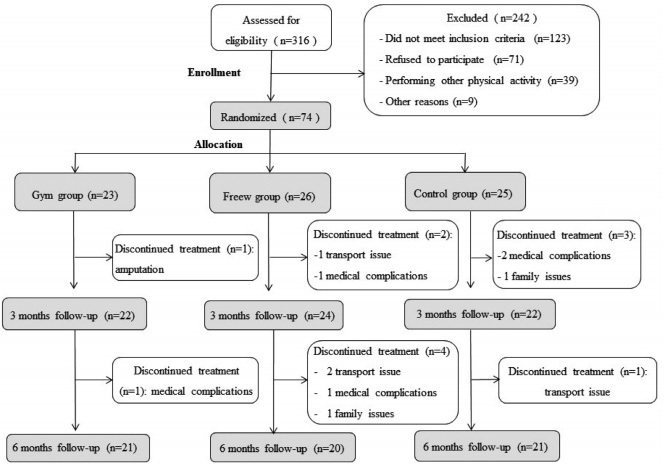
The CONSORT flow diagram.

The participants were randomly assigned to one of three groups: RT by using weightlifting machines at a gym (gym group); RT by using free weights and elastic bands (freew group); and a control group. We used a computer random number generator to create 13 blocks of six-number sequences (expecting a study dropout proportion of 20% or more). Randomly generated number sequences were placed in sealed opaque envelopes and randomly assigned to patients after enrollment. A physiotherapist, blinded to intervention assignment, examined all participants before and after the intervention in their best clinical condition (ON state). The flow chart shows group allocation (Figure 1).

*Consolidated Standards of Reporting Trials*: patients recruitment and follow-up. Randomized, single-blinded clinical trial with three groups: Gym: resistance training with gym equipment group; FreeW: resistance training with free weights group; Control: control group.

### Study intervention

The conceptual framework of our intervention was based on the American College of Sports Medicine guidelines^19^. It is recommended that free-weight multiple machines and single-joint exercises should be used. For older patients, the lifting velocity should be slow to moderate, with one to three sets per exercise, at 60-80% of a one-repetition maximum (1-RM) for 8-12 repetitions with 1-3 min of rest between sets^19^.

A group of up to four patients participated in each RT session, consisting of 50 minutes of training, twice a week for 3 months. The aim of both RT groups was to activate all postural muscles, especially trunk muscles, that play a role in maintaining balance during motor performance and in reducing the risk of falls^20^^,^^21^. Lower-limb muscles were also recruited for stability in performing the exercises.

Each RT session started with a five-minute warm-up, in which the participants were asked to side-tilt and rotate the trunk with their arms abducted, and to raise and lower their arms without moving the trunk. They were then instructed to perform hip flexion, extension and abduction with 10 repetitions of each exercise. At the end of each session, there was a cool-down period that included upright stretching of quadriceps, hamstring, triceps brachii and pectoris muscles for 15 seconds each^22^.

We chose to perform two different RT protocols due to lack of evidence regarding what the most effective type of exercise might be, for improving postural control in PD. All three groups were instructed to perform stretching exercises at home.

#### RT by using weightlifting machines at a gym (gym group)

**Figure 2.**
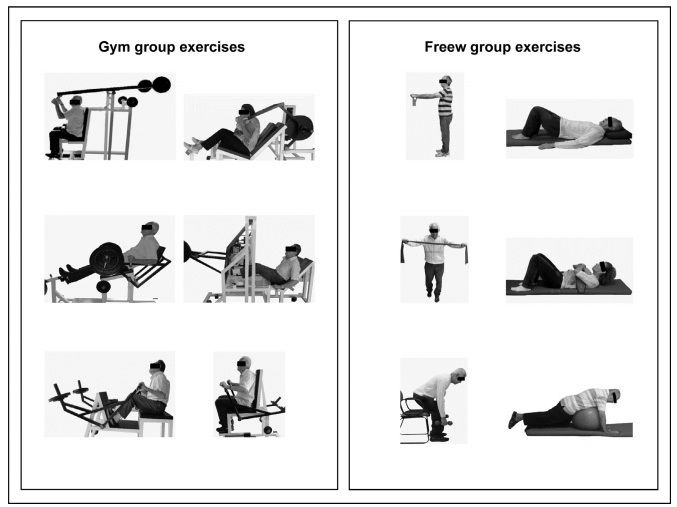
Resistance training program.

The participants performed RT using weightlifting machines at a gym (Biodelta®, São Paulo, Brazil). The initial workload was defined as 60% of a one-repetition maximum (1-RM) and then they were encouraged to perform three sets of 8 to 12 repetitions with 60-second rests between sets^19^. The workload was progressively increased by 5 to 10% if the patient did not feel muscle fatigue after the previous training^19^. The weightlifting exercises included lateral pulldown, back extension, seated row, seated chest press, abdominal crunch and leg press (Figure 2).

Participants in the gym group performed resistance exercises using machines, which included lateral pulldown, back extension, seated row, seated chest press, abdominal crunch and leg press. The main objective was to recruit postural muscles, especially extensor trunk muscles with lower-limb stabilization to perform each exercise. Workloads were progressively increased by 5 to 10%. Participants in the freew group performed exercises aimed at recruiting the same muscle groups as in the gym group, which were: abdominals, paraspinal, middle trapezius, latissimus dorsi, rhomboid, quadriceps femoris, gluteal muscles and lower limbs. The training workload was increased through use of dumbbells, elastic bands and ankle weights. All participants were instructed not to perform the Valsalva maneuver during the movement phase, but to activate abdominal muscles during expiration.

#### RT by using free weights and elastic bands (freew group)

The RT program targeted the same muscle groups as in the gym group, including abdominal, paraspinal, middle trapezius, latissimus dorsi, rhomboid, quadriceps femoris and gluteal muscles. The workload was progressively increased, using dumbbells, elastic bands and ankle weights if the patient did not feel muscle fatigue after the previous training^19^ (Figure 2).

#### Control group

Each participant received a booklet describing sets of stretching exercises to be performed twice a week during the study period. They were instructed to perform a variety of seated and standing 15-second stretches involving trunk, hamstring, pectoral, brachial triceps and quadriceps muscles^23^. Their practice frequency was monitored through phone calls. This exercise protocol is customarily used in rehabilitation programs and has no equivalent workloads in RT.

### Primary endpoint measurements

The study was conducted at the Laboratory of Movement Study, Instituto de Ortopedia e Traumatologia, Hospital das Clínicas, Faculdade de Medicina, Universidade de São Paulo, Brazil.

For SP, the participants were placed in a quiet upright stance on the force platform (AccuSway Plus, Advanced Mechanical Technology Inc., AMTI, Massachusetts, United States). Postural sway data was analyzed using the Balance Clinic® software. They were instructed to maintain a comfortable standing position and look fixedly at a spot one meter away and not to move or speak during the test (unless when performing the dual-task condition). A baseline support base was drawn on a sheet of paper, for use in subsequent assessments. The mean measurements for three 60-second trials were recorded for each condition tested: eyes-open (EO), eyes-closed (EC) and dual-task (DT). For the latter, the participants were asked to say as many words beginning with the letter F as possible, during the whole test period, and then to name as many animals and fruits as they could. The primary outcome measurements included the following COP displacement variables assessed in SP after the RT intervention:

- Mediolateral displacement (ML), representing the standard deviation of the COP on the mediolateral axis, expressed in centimeters (cm).
- Anteroposterior displacement (AP), representing the standard deviation of the COP on the anteroposterior axis, expressed in cm.
- Velocity, as the mean velocity of COP displacement in all directions, measured in centimeters per second (cm/s).
- Area of the ellipsis that covers 95% of the COP trajectory, expressed in square centimeters (cm^2^).

### Secondary endpoint measurements

The secondary outcome measurements of the study included: Unified Parkinson's Disease Rating Scale, Part III (motor examination) (UPDRS-III); Berg Balance Scale (BBS); Mini-Balance Evaluation Systems Test (Mini-Best); Timed Up and Go (TUG) test to assess functional balance; 39-item Parkinson's Disease Questionnaire (PDQ-39) to assess how often people affected by Parkinson's disease experience difficulties across eight dimensions of daily living: mobility, impact on activities of daily living, bodily discomfort, emotional wellbeing, stigma, social support, cognition and communication domains; and dynamic posturography (Balance Master platform, NeuroCom® International Inc., Oregon, United States) to assess the following tasks: a) stepping up and over an obstacle: first stepping with the left leg then swinging the opposite leg onto a 10-cm-high box and then landing the left leg on the force plate. The leg lift-up index quantifies the maximal lifting force exerted by the leading leg and is expressed as a percentage of the individual’s body weight; b) movement time (MovTime) quantifies the number of seconds required to complete the task of stepping up and over an obstacle; and c) tandem speed is the velocity at which tandem walking is performed, expressed as cm/s. The score recorded was the mean value of three trials for each task.

All measurements were collected at baseline, at one week after completing the intervention period (at three months) and at the six-month follow-up.

### Statistical analysis

The sample size was calculated after a pilot study. The number of participants required to detect a change of at least one standard deviation in SP measurements was 21 for each group (power=0.8; alpha=0.05).

Differences in baseline characteristics among groups were assessed by means of univariate analysis of variance (ANOVA) for age, MMSE scores and disease duration. We also tested for the equality-of-proportion hypothesis, for HY stage, gender and race/ethnic group.

To determine the effect of treatment, two-way ANOVA (group versus time) was used to compare posturography measurements and functional balance scores. Whenever an interaction was noted, Tukey’s multiple-comparison *post-hoc* test was used to compare each pair of groups for each outcome.

All analyses were conducted on an intention-to-treat basis using the Statistica software package v. 13.3 (TIBCO, United States) and Excel Office 2010. An α level of significance was set at p<0.05 and all tests were two-sided.

## RESULTS

Out of 316 patients screened for eligibility, 74 met the inclusion criteria and were enrolled in the study. Six patients did not complete the training protocol (Figure 1).

### Baseline characteristics

Table 1 shows the demographic and clinical characteristics of the participants. There were no significant differences among the groups at baseline with regard to MMSE, UPDRS-III, Mini-Best and BBS, TUG, PDQ-39 domains scores or posturography variables (p>0.05).

**Table 1.** Demographic and clinical characteristics of the participants.

	Gym group (n=23)	Freew group (n=26)	Control (n=25)	Gym group vs. control p-value	Freew group vs. control p-value	Gym group vs. Freew group p-value	p-value
Gender, n (%)							
Male	17 (73.9)	18 (69.2)	18 (72)	0.882ᵃ	0.828ᵃ	0.717ᵃ	_
Female	6 (26.1)	8 (30.8)	7 (28)				
Race/ethnic group, n (%)							
White	16 (69.6)	17 (65.4)	13 (52)	0.214ᵃ	0.332ᵃ	0.755ᵃ	_
Black	1 (4.3)	0	2 (8)	0.602ᵃ	0.141ᵃ	0.283ᵃ	
Mixed	5 (21.7)	7 (26.9)	8 (32)	0.424ᵃ	0.691ᵃ	0.674ᵃ	
Asian	1 (4.3)	2 (7.7)	2 (8)	0.602ᵃ	0.967ᵃ	0.626ᵃ	
HY stage, n (%)							
2	6 (26.1)	3 (11.5)	6 (24)	0.868ᵃ	0.243ᵃ	0.189ᵃ	_
2.5	14 (60.9)	20 (76.9)	16 (64)	0.823ᵃ	0.311ᵃ	0.224ᵃ	
3	3 (13)	3 (11.5)	3 (12)	0.913ᵃ	0.959ᵃ	0.873ᵃ	
Education level, years							
Mean (SD)	7.3 (5.1)	9.4 (4.4)	8.5 (3.8)	_	_	_	0.255ᵇ
Range	2-19	2-16	3-15				
BMI							
Mean (SD)	25.6 (3.1)	25.9 (3.6)	25.7 (4.3)	_	_	_	0.972ᵇ
Range	18.5-33.6	19.4-32.5	18.3-36.5				
Age, years							
Mean (SD)	63.4 (6.9)	63.2 (6.4)	63.6 (7)	_	_	_	0.977ᵇ
Range	50-75	50-74	52-75				
Disease duration, years							
Mean (SD)	7.6 (6)	8.4 (5.9)	9.6 (4.8)	_	_	_	0.462ᵇ
Range	2-30	2-25	2-18				
MMSE scores							
Mean (SD)	27.4 (1.9)	26.9 (2.4)	27.5 (2.1)	_	_	_	0.527ᵇ
Range	24-30	24-30	24-30				

Data presented as mean (standard deviation, SD) or (%). %: percentage; N: number; HY stage: Hoehn and Yahr stage; BMI: body mass index; MMSE: Mini-Mental State Examination; ᵃ: test for equality of proportions, ᵇ: ANOVA.

### Effects of intervention

**Figure 3.**
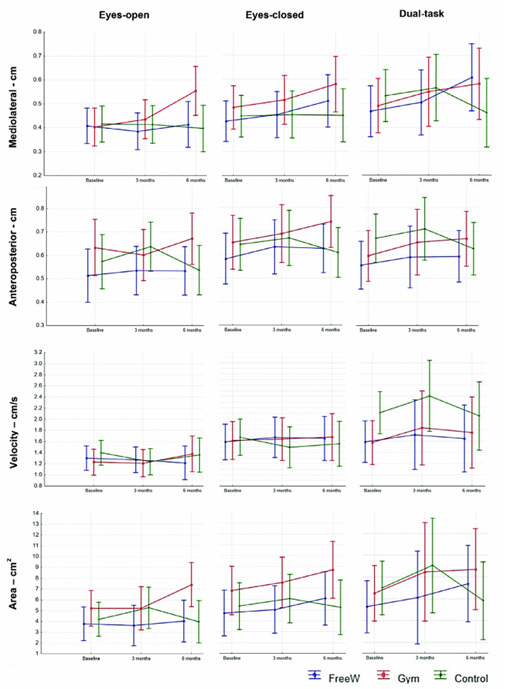
Static posturography measurements under eyes-open, eyes-closed and dual-task conditions

ANOVA did not show any significant group versus time interactions in SP measurements (primary outcome), in relation to the eyes-open condition for ML (F_4,142_=2.232; p=0.068), AP (F_4,142_=2.125; p=0.080), velocity (F_4,142_=0.615; p=0.666) or area (F_4,142_=2.021; p=0.094). Similarly, there was no significant main effect regarding group in SP measurements, in relation to the eyes-closed condition for ML (F_4,142_=0.747; p=0.561), AP (F_4,142_=1.582; p=0.182), velocity (F_4,142_=0.386; p=0.817) or area (F_4,142_=0.758; p=0.553). There was also no significant effect regarding the dual-task condition for ML (F_4,142_=1.652; p=0.164), AP (F_4,142_=0.640; p=0.634), velocity (F_4,142_=0.192; p=0.941) or area (F_4,142_=0.755; p=0.556) (Figure 3).

ANOVA showed that there was a group-versus-time interaction for UPDRS-III scores (F_4,142_=3.396; p=0.010). Tukey’s *post-hoc* tests showed a reduction in UPDRS-III score at the three-month follow-up, compared with baseline (26.46 vs. 29.58; p=0.028), in the freew group and at the three-month follow-up, compared with baseline (25.61 vs. 29.13; p=0.014), in the gym group (Table 2).

**Table 2.** Functional clinical tests, dynamic posturography and quality of life.

	Gym group			Freew group			Control group			P value
	Baseline	3 months	6 months	Baseline	3 months	6 months	Baseline	3 months	6 months	
UPDRS-III (0-108)	29.13 (10.06)	25.61 (10.03)	27.65 (9.92)	29.58 (12.06)	26.46 (11.17)	28.38 (10.05)	26.44 (9.95)	27.48 (7.99)	27.60 (8.12)	0.010*
TUG (sec)	8.70 (3.39)	8.04 (3.27)	7.91 (2.89)	8.5 (2.10)	7.88 (1.88)	7.96 (1.93)	8.56 (1.73)	8.20 (1.87)	8.12 (1.88)	0.894
BBS (0-56)	52.09 (4.5)	53.17 (3.17)	52.96 (2.93)	51.00 (4.74)	52.62 (3.02)	52.96 (2.82)	52.28 (2.79)	52.28 (3.41)	52.24 (3.07)	0.043*
Mini-Best (0-32)	24.48 (4.24)	25.87 (4.97)	25.70 (4.24)	23.69 (4.71)	25.35 (4.04)	25.69 (3.92)	24.92 (4.14)	24.52 (3.97)	25.04 (3.66)	0.014*
Stepping up										
Lift-up index left (%)	16.65 (5.25)	19.74 (5.75)	19.04 (3.96)	16.35 (5.59)	19.00 (6.89)	18.65 (7.53)	19.48 (8.28)	19.08 (6.32)	20.84 (6.84)	0.253
Lift-up index right (%)	18.48 (7.48)	20.48 (7.48)	20.17 (6.04)	16.69 (7.00)	19.77 (8.65)	18.88 (8.38)	18.00 (6.81)	19.04 (7.04)	20.76 (7.22)	0.583
MovTime left (sec)	2.02 (0.71)	1.81 (0.65)	1.69 (0.55)	2.09 (0.73)	1.92 (0.78)	1.96 (0.73)	1.82 (0.40)	1.82 (0.38)	1.70 (0.30)	0.232
MovTime right (sec)	1.93 (0.70)	1.69 (0.64)	1.76 (0.77)	1.95 (0.07)	1.80 (0.68)	1.85 (0.77)	1.71 (0.39)	1.68 (0.30)	1.63 (0.35)	0.613
Tandem speed (cm/s)	19.40 (11.4)	20.93 (10.37)	22.58 (11.63)	19.80 (5.96)	22.65 (7.16)	22.00 (7.91)	19.38 (5.73)	19.25 (6.20)	19.82 (5.54)	0.132
PDQ-39										
Mobility	34.72 (24.10)	21.46 (21.04)	25.80 (25.16)	30.52 (22.02)	23.94 (19.34)	24.63 (20.63)	23.12 (19.51)	24.94 (17.64)	23.52 (17.97)	0.019*
Daily living	37.58 (23.70)	25.21 (19.53)	29.21 (19.61)	36.53 (25.99)	28.21 (21.14)	29.02 (22.18)	24.24 (21.44)	24.03 (16.41)	20.18 (16.55)	0.247
Emotional wellbeing	30.79 (22.68)	21.04 (16.54)	22.45 (17.73)	28.54 (23.44)	22.61 (15.08)	27.43 (16.35)	22.36 (16.65)	22.68 (15.42)	23.70 (17.41)	0.304
Stigma	20.90 (21.62)	19.03 (22.65)	14.10 (17.21)	25.03 (18.38)	19.73 (18.17)	18.78 (16.79)	12.78 (15.89)	13.49 (18.46)	11.52 (15.71)	0.749
Social Support	15.78 (16.84)	9.06 (13.68)	10.14 (15.26)	15.88 (22.87)	11.54 (22.74)	11.22 (19.14)	8.19 (13.95)	9.33 (15.07)	12.14 (18.85)	0.373
Cognition	26.50 (20.87)	25.30 (17.55)	24.25 (19.03)	27.44 (20.36)	27.39 (19.60)	26.46 (20.23)	19.52 (19.30)	18.78 (15.62)	17.00 (18.84)	0.997
Communication	26.19 (20.64)	22.49 (19.56)	21.69 (18.11)	26.28 (26.22)	23.65 (19.49)	20.86 (15.54)	27.49 (17.99)	23.97 (16.36)	25.02 (21.37)	0.954
Bodily discomfort	42.43 (24.66)	26.78 (22.02)	32.16 (20.35)	25.96 (26.01)	25.47 (14.05)	25.92 (20.19)	34.97 (21.89)	32.32 (20.87)	32.35 (17.70)	0.078

Data presented as mean (standard deviation), sec: seconds; %: percentage; cm/s: centimeters per second; UPDRS-III: Unified Parkinson’s Disease Rating Scale, part III; TUG: timed up & go; MovTime: movement time; PDQ-39: quality of life perception; BBS: Berg balance scale; Mini-Best: Mini-Balance Evaluation Systems Test; lift-up index: maximal lifting force; Tandem speed: tandem walk speed; *: difference among groups (p<0.05).

For Mini-Best scores, a significant group-versus-time interaction was observed (F_4,142_=3.231; p=0.014). Tukey’s *post-hoc* tests showed improved scores at the three-month (25.35 vs. 23.69; p=0.015) and six-month follow-ups, compared with baseline (25.69 vs. 23.69; p=0.001), in the freew group only.

Similarly, a significant group-versus-time interaction was observed for BBS scores (F_4,142_=2.529; p=0.043). Tukey’s *post-hoc* tests showed score improvements at the three-month (52.62 vs. 51.00; p=0.020) and six-month follow-ups, compared with baseline (52.96 vs. 51.00; p=0.001), in the freew group.

For PDQ-39 domains, group-versus-time interaction was seen for the mobility domain (F_4,142_=3.021; p=0.019). Tukey’s *post-hoc* test showed score improvement at the three-month follow-up, compared with baseline (21.46 vs. 34.72; p=0.001) in the gym group. Interactions were not significant for other domains: activities of daily living (F_4,142_=1.368; p=0.247); bodily discomfort (F_4,142_=2.144; p=0.078); emotional well-being (F_4,142_=1.220; p=0.304); stigma (F_4,142_=0.637; p=0.636); social support (F_4,142_=1.070; p=0.373); cognition (F_4,142_=0.346; p=0.997); and communication (F_4,142_=0.166; p=0.954) (Table 2).

For TUG, no group-versus-time interaction was seen in any group (F_4,142_=0.273; p=0.894) (Table 2).

For dynamic posturography, no significant group-versus-time interaction was seen for tandem walking speed task (F_4,142_=1.800; p=0.132). Similarly, for the task of stepping up and over an obstacle, there was no significant interaction for lift-up index starting with the left leg (F_4,142_=1.351; p=0.253) or the right leg (F_4,142_=0.798; p=0.528). Likewise, no interaction for movement time was seen for the left leg (F_4,142_=1.414; p=0.232) or the right leg (F_4,142_=0.670; p=0.613) (Table 2).

There were no serious adverse events in our study. The events reported during training sessions for the freew group included a fall episode (one participant), mild transient joint pain (three participants) and orthostatic hypotension (three participants), with no serious injury. For the gym group, there were reports of an outdoor fall (one participant), mild transient muscle pain (three participants) and orthostatic hypotension (two participants). For the control group, only one participant reported joint pain.

## DISCUSSION

Our study found that there were no statistically significant changes in SP measurements following a three-month RT intervention among PD patients. However, there is no consensus on SP measurements and how they correlate with postural control in PD patients.

Some authors have suggested that larger COP displacement is likely to be a predictor of postural instability^8^^,^^24^, but few have investigated SP measurement after RT in PD. Santos et al.^25^ assessed the effects of two months of RT using gym weightlifting equipment, starting at a workload of 40% of 1-RM. In addition to improved gait speed, they found only a reduction in COP sway path length measurements after the training^25^. Similarly, a ten-week high-intensity RT intervention resulted in a 29% increase in the posterior COP sway and 11% increase in COP velocity^26^. According to those authors, larger posterior COP sway could increase arm movement by shifting the center of gravity forward and helping gait initiation, which suggests that lower-limb training may interfere with anticipatory postural adjustments^26^. However, the sample size was small and these findings should be interpreted with caution. In contrast, another three-month RT protocol including trunk and lower limb exercises did not show any changes in COP sway, in comparison with balance exercises^27^. Although this balance program gave rise to improvements in clinical balance tests, these were insufficient to show on SP measurements^27^.

Although the dual-task condition has been shown to influence postural control in PD^5^, we did not see this effect in our patients. Despite methodological differences between the RT intervention evaluated in our study and those of the other studies mentioned above, it is important to point out that the question remains whether one or two COP variable changes after a RT program can be inferred to represent a functional gain in PD patients. SP is considered to be the gold standard for the evaluation of postural control, but postural instability is multifactorial in PD and muscle strengthening alone may be insufficient to improve postural adjustments so as to maintain an upright stance.

Our RT protocol did not have any impact on dynamic posturography measurements and the same question can be raised as in relation to SP. Although the PD patients were 25% weaker and slower in lifting their leg over a box, compared with healthy controls^28^, studies have found no improvement in postural parameters after three^29^ or six-month RT interventions^23^, and the intervention was insufficient to optimize strategies for gains in functional independence^12^.

In our study, significant improvement in motor symptoms was seen in both RT groups, with reductions in UPDRS-III scores (-3.52 for the gym group; -3.12 for the freew group). In contrast, these scores increased in the control group (+1.04). Previous studies showed that a score reduction of 2.3 to 2.7 is clinically relevant^30^. One study reported that a reduction in UPDRS-III score was maintained up to a 24-month follow-up^13^ and another demonstrated a score reduction of 5.07 after a six-month RT intervention^23^. However, there was no change in UPDRS scores after a two-month RT intervention; its short duration and/or training design were insufficient to promote neuromuscular adaptations^25^.

The impact of RT on functional mobility is not yet clear. TUG time was reduced after three-month^31^ and six-month RT programs^23^. In contrast, our findings concur with the results from a meta-analysis that reported that RT was not superior to other training interventions regarding TUG time^32^. Likewise, another three-month RT intervention increased muscle strength, but was insufficient to increase TUG time^10^. None of the studies mentioned above reported any reduction close to 3.5 seconds, which is considered to be the minimal clinically significant difference^33^. It is noteworthy that the three groups in our study showed good time performance at baseline (less than 9 seconds), so it is possible that a ceiling effect may have occurred.

The freew group performed better in the BBS and Mini-Best tests. Although BBS is a widely used scale, ceiling effects are likely to occur. Thus the Mini-Best scale is more sensitive for detecting postural instability than BBS^34^. Bearing in mind that a three-month high-intensity RT intervention had a positive impact on BBS scores^35^ and another three-month RT protocol did not improve on BESTest scores^27^, we chose to use both scales in order to broaden our functional assessment. The freew group was asked to perform more coordinated specific sequences of movements against different external loads and, even though they trained at a lower workload than the gym group, this practice may have been more demanding in terms of motor control and may have resulted in better postural control.

In our study, we found better perceived QoL for the mobility domain (PDQ-39) in the gym group after RT intervention. Our findings are in accordance with those of other studies reporting better perceived QoL following a two-month high-intensity training program^25^ and a six-month program^13^.

Our study had some limitations. We cannot rule out the existence of a placebo effect since the control group could have expected to participate in RT intervention; the isokinetic machine for muscle strengthening was not available for our study; the participants in our sample were not stratified for the presence of dyskinesia, and involuntary movements may have influenced posturographic measurements and may have had a confounding effect or produced outliers; and our training protocol did not include exercises for plantar flexion and dorsiflexion or for hip abduction or adduction, which may have influenced our balance measurements, especially static balance. However, the strengths of this study were the single-blind randomized design with a supervised training protocol and the three-month follow-up after the intervention.

Although SP allows quantitative measurements of body sway, our findings suggest that it was not an appropriate tool for discriminating postural control changes after RT intervention and it may have limited value in assessing patients in clinical practice. SP is considered to be the golden standard assessment for postural control measurements, but in our study the functional scales provided better assessments on the functional capacity of our sample of patients. Therefore, further studies with more comprehensive assessments of the impact of RT and posturography measurements are needed.

The study intervention helped to improve motor ability and perceived QoL in the gym group. It helped to improve motor and balance scores with moderately positive effects in the freew group, possibly because training with free weights required greater postural motor control. Both protocols were well accepted and could easily be implemented in centers for physical activities. Overall, there was good adherence to training among the participants and they showed no serious adverse events during the exercise sessions, which suggests that this training was safe.

In conclusion, after this three-month training intervention, there were no changes in SP measurements. However, both intervention groups showed improved motor performance (UPDRS-III motor scores) with better perceived QoL in the gym group and moderate effects on functional balance in the freew group.
